# Blunted Endogenous Opioid Release Following an Oral Amphetamine Challenge in Pathological Gamblers

**DOI:** 10.1038/npp.2015.340

**Published:** 2015-12-09

**Authors:** Inge Mick, Jim Myers, Anna C Ramos, Paul R A Stokes, David Erritzoe, Alessandro Colasanti, Roger N Gunn, Eugenii A Rabiner, Graham E Searle, Adam D Waldman, Mark C Parkin, Alan D Brailsford, José C F Galduróz, Henrietta Bowden-Jones, Luke Clark, David J Nutt, Anne R Lingford-Hughes

**Affiliations:** 1 Centre for Neuropsychopharmacology, Division of Brain Sciences, Faculty of Medicine, Imperial College London, London, UK; 2 Department of Psychobiology, Universidade Federal de São Paulo, São Paulo, Brazil; 3 Centre for Affective Disorders, Department of Psychological Medicine, Institute of Psychiatry, Psychology and Neuroscience, King's College London, London, UK; 4 Imanova Ltd., Centre for Imaging Sciences, London, UK; 5 Department of Neuroimaging, Institute of Psychiatry, King's College, London, UK; 6 Department of Imaging, Division of Experimental Medicine, Department of Medicine, Imperial College, London, UK; 7 Drug Control Centre, Analytical and Environmental Sciences, King's College London, London, UK; 8 National Problem Gambling Clinic, CNWL NHS Foundation Trust, Imperial College London, London, UK; 9 Centre for Gambling Research, Department of Psychology, University of British Columbia Vancouver, Vancouver, Canada

## Abstract

Pathological gambling is a psychiatric disorder and the first recognized behavioral addiction, with similarities to substance use disorders but without the confounding effects of drug-related brain changes. Pathophysiology within the opioid receptor system is increasingly recognized in substance dependence, with higher mu-opioid receptor (MOR) availability reported in alcohol, cocaine and opiate addiction. Impulsivity, a risk factor across the addictions, has also been found to be associated with higher MOR availability. The aim of this study was to characterize baseline MOR availability and endogenous opioid release in pathological gamblers (PG) using [^11^C]carfentanil PET with an oral amphetamine challenge. Fourteen PG and 15 healthy volunteers (HV) underwent two [^11^C]carfentanil PET scans, before and after an oral administration of 0.5 mg/kg of d-amphetamine. The change in [^11^C]carfentanil binding between baseline and post-amphetamine scans (ΔBP_ND_) was assessed in 10 regions of interest (ROI). MOR availability did not differ between PG and HV groups. As seen previously, oral amphetamine challenge led to significant reductions in [^11^C]carfentanil BP_ND_ in 8/10 ROI in HV. PG demonstrated significant blunting of opioid release compared with HV. PG also showed blunted amphetamine-induced euphoria and alertness compared with HV. Exploratory analysis revealed that impulsivity positively correlated with caudate baseline BP_ND_ in PG only. This study provides the first evidence of blunted endogenous opioid release in PG. Our findings are consistent with growing evidence that dysregulation of endogenous opioids may have an important role in the pathophysiology of addictions.

## INTRODUCTION

Pathological gambling (PG) is a psychiatric disorder characterized by a pre-occupation with thoughts of gambling, repeated attempts to reduce or quit, debt and/or illegal activity, and disruption of personal relationships and/or employment. PG has been estimated to affect between 0.2 and 5.3% of the adult population worldwide (Hodgins *et al*, 2011). Originally classified as an ‘Impulse Control Disorder' in DSM-IV, PG has recently been reconceptualized as a ‘behavioral addiction' (Bowden-Jones and Clark, 2011), now classified as a ‘Substance-Related and Addictive Disorder' in DSM-5 due to the observed similarities with substance addiction. These include clinical and etiological features such as abnormal reward sensitivity (van Holst *et al*, 2010), disadvantageous decision making and diminished behavioral inhibition (Verdejo-Garcia *et al*, 2008). PG constitutes a useful model to provide broader insights into the core brain processes of addictive disorders as it does not involve the confounding effects of excessive and chronic substance use on brain function.

Psychological treatments including cognitive behavioral therapy (CBT) are currently the first-line therapies for PG; however, these are associated with only a partial response and short-term benefits (Cowlishaw *et al*, 2012). There has been less exploration of the neuropharmacology of PG, despite evidence from preliminary trials that opiate antagonists may be beneficial (Potenza, 2008). There is a wealth of evidence indicating a key role for the opioidergic system in substance dependence, as well as in related constructs such as reward and impulsivity. The opiate antagonists, naltrexone and nalmefene, are also proven treatments for addictions, particularly alcoholism (Lingford-Hughes *et al*, 2012). Endogenous opioids in the brain comprise a number of peptides (β-endorphins, dynorphins, enkephalins) and their receptors (mu, kappa, delta, respectively) are widely distributed throughout the brain. Mu-opioid receptors (MOR) have a key role in mediating rewarding effects of opiates and are most dense in the basal ganglia, thalamus, and amygdala (Mansour *et al*, 1988).

Evidence of a dysregulated opioid system in alcohol, cocaine, and opiate addiction has been shown using positron emission tomography (PET) with the selective MOR agonist radioligand [^11^C]carfentanil, which has greater selectivity of the order 100-fold for MOR over other subtypes (Subramanian *et al*, 2000; Titeler *et al*, 1989) or the non-selective tracer [^11^C]diprenorphine. In these studies, higher MOR availability is consistently reported (Gorelick *et al*, 2005; Heinz *et al*, 2005; Williams *et al*, 2007, 2009; Zubieta *et al*, 1996). Higher MOR availability is also associated with craving, which might contribute to the high rates of relapse during early abstinence (Gorelick *et al*, 2005). It has therefore been argued that as higher MOR availability is seen across addictions to substances with differing pharmacology, these changes are fundamental to addiction rather than substance-specific (Williams *et al*, 2009). Higher MOR availability, measured with [^11^C]carfentanil PET may reflect either an increase in receptor density or a reduction in endogenous *β*-endorphin levels to which [^11^C]carfentanil is sensitive (Colasanti *et al*, 2012; Mick *et al*, 2014). A reduction in endogenous *β*-endorphin levels would be consistent with the concept that addiction vulnerability is associated with an ‘opioid-deficient' state, which is compensated for by drug taking (Ulm *et al*, 1995). Consistent with this hypothesis, individuals with an elevated risk of alcoholism, and a heightened response to alcohol, have been shown to have lower basal levels of plasma *β*-endorphin (Gianoulakis, 1996).

Previously, we have shown in two independent healthy participant (HV) cohorts that an oral d-amphetamine (0.5 mg/kg) challenge releases endogenous opioids as indicated by a reduction in [^11^C]carfentanil binding (Colasanti *et al*, 2012; Mick *et al*, 2014). This approach allows us for the first time to directly measure opioid release *in vivo* in the brain of individuals with addictive disorders. In the present study, we used this technique to examine both baseline MOR availability and endogenous opioid release in PG. Previous studies using [^11^C]carfentanil have shown excellent reproducibility of [^11^C]carfentanil-binding parameter estimates (Hirvonen *et al*, 2009). Given the similarities in behavior between PG and substance addiction, we hypothesized that PG would be associated with higher baseline MOR levels. On the basis of the ‘opioid-deficient' hypothesis in alcoholism, we hypothesized that endogenous opioid release after amphetamine in PG would be attenuated.

## MATERIALS AND METHODS

### Participants

Fifteen males with PG were recruited from the National Problem Gambling Clinic, Central North West London NHS Foundation Trust, UK. One participant's PET data were not quantifiable owing to technical issues, leaving 14 PG (mean age±SD 34.3±7.65 years, three smokers). Fifteen male age-matched HV (34.5±8.77 years, two smokers) have been previously described in our original (Colasanti *et al*, 2012) six who received ‘high dose' amphetamine and second cohort (Mick *et al*, 2014). Only men were studied owing to the small number of women in treatment at the clinic, at 7% of clinic attendees. Participants' current/previous medical/mental health as well as history of alcohol, tobacco, and other substance use were assessed by trained psychiatrists using Mini Psychiatric Interview International (MINI-5) (Sheehan *et al*, 1998). HV with current or previous psychiatric disorders were excluded. In PG, past depression and anxiety was allowed, as these are common comorbidities; current depression or anxiety was excluded. Current or past history of substance abuse or dependence including alcohol but except nicotine, was an exclusion criterion; previous recreational drug use was allowed (>10 times in lifetime: one HV: cannabis; two PG: cannabis and cocaine). Participants were excluded if they drank more than 21 UK units of alcohol (166 g) per week. Other drug use (except tobacco) was not allowed 2 weeks prior and during the study. This was confirmed on study days by negative urine drug screen testing (cocaine, amphetamine, THC, methadone, opioids, benzodiazepines) and participants also breathalyzed negative for alcohol. Smoking was not allowed 1 h before each scan. All the participants had laboratory and ECG results within normal range; none were taking regular medication.

PG were recruited either before or during an 8-week course of CBT and all had a recent history of active gambling; ‘days of abstinence' ranged between 3 and 128 days (mean±SD 47±40.8). DSM-IV diagnosis of PG was confirmed with the Massachusetts Gambling Screen (Shaffer *et al*, 1994; MAGS; mean±SD 7±1.9) corroborated by the Problem Gambling Severity Index (Ferris and Wynne, 2001; PGSI; mean±SD 18±5.2). The Gambling Craving Scale (Young and Wohl, 2009; GACS) measured baseline craving for gambling on the study day. Depression was assessed with the Beck Depression Inventory (BDI) and anxiety with Spielberger Trait inventory (STAI). To assess impulsivity, the UPPS-P Impulsive Behavior Scale (Cyders *et al*, 2007) was used with its five subscales: Negative Urgency (NU), Positive Urgency (PU), Lack of Planning (LoP), Lack of Perseverance (LoPe), and Sensation Seeking (SS).

On the screening day, participants underwent structural and functional magnetic resonance imaging (MRI) and performed a computerized neurocognitive assessment; these results will be reported elsewhere.

Written informed consent was obtained from all the participants. The study was approved by the West London Research Ethics Committee and the Administration of Radioactive Substances Advisory Committee, UK.

### Procedure

PET imaging procedures were identical to our previous studies with [^11^C]carfentanil (Colasanti *et al*, 2012; Mick *et al*, 2014). Briefly, participants underwent two [^11^C]carfentanil PET scans, one before and one 3 h following an oral administration of 0.5 mg/kg of d-amphetamine. Nine HV underwent both PET scans on the same day. For six HV, the post-amphetamine scan was acquired on a different day for logistic reasons; none of them received two doses of amphetamine. The average time between pre- and post scans in these cases was 8 days (range: 1–36 days). For PG, 13 out of 14 participants had their pre- and post-amphetamine PET scans on the same day. One PG participant had his scans 8 days apart. The oral d-amphetamine was administered 3 h before the post- amphetamine scan, after a light meal, based upon peak amphetamine plasma levels (Mick *et al*, 2014). Blood samples to assess plasma levels were obtained pre-dosing; 1; 2; 3, and 4.5 h post dosing.

Subjective responses to the amphetamine challenge were rated using the simplified version of the Amphetamine Interview Rating Scale (SAIRS) (Van Kammen and Murphy, 1975), consisting of self-ratings for euphoria, restlessness, alertness, and anxiety (from 1 (least ever felt) to 10 (most ever felt)). It was administered after the pre-amphetamine scan, 15 min pre-dosing and post dosing at 5 min, 1, 2, and 3 h (just before the post-amphetamine scan) and 4.5 h.

### PET and MR Imaging

As previously (Colasanti *et al*, 2012; Mick *et al*, 2014), dynamic [^11^C]carfentanil PET scans were acquired on a HiRez Biograph 6 PET/CT scanner (Siemens Healthcare, Erlangen, Germany). Dynamic emission data were collected continuously for 90 min (26 frames, 8 × 15 s, 3 × 60 s, 5 × 120 s, 5 × 300 s, 5 × 600 s), following an intravenous injection of 217±66.07 (mean±SD) MBq of [^11^C]carfentanil in HV and 211±58.42 MBq in PG. All the participants underwent a T1-weighted structural MRI (Magnetom Trio Syngo MR B13 Siemens 3T; Siemens AG, Medical Solutions). All the structural images were reviewed by an experienced neuroradiologist for unexpected findings of clinical significance. None were observed; however, one PG participant was excluded owing to the inaccurate spatial normalization of PET data into standard space using the structural MR data.

### Image Analysis

As described previously (Colasanti *et al*, 2012; Mick *et al*, 2014), pre-processing of images and PET modeling were carried out using MIAKAT, an analysis tool developed at Imanova. After frame-by-frame motion correction of the dynamic PET data, region-of-interest (ROI) time-activity data were sampled using the CIC neuroanatomical atlas (Tziortzi *et al*, 2011). This was applied to the PET image by non-linear deformation parameters derived from the transformation of the structural MRI into standard space.

Nine grey-matter-masked ROIs were chosen *a priori*, as brain areas with a high density of MOR, with significant amphetamine-induced reductions of [^11^C]carfentanil BP_ND_, and relevant to addiction—the caudate, putamen, thalamus, cerebellum, frontal lobe, nucleus accumbens, anterior cingulate, amygdala, and insula cortices. A tenth region of interest, hypothalamus, where endorphin cell bodies are located, was manually defined on the MRI of each subject according to anatomical reference as described previously (Colasanti *et al*, 2012; Tziortzi *et al*, 2011). BP_ND_ was quantified regionally using the simplified reference tissue model with occipital lobe as the reference region (Colasanti *et al*, 2012). Endogenous opioid release was indexed as the fractional reduction in [^11^C]carfentanil BP_ND_ following the d-amphetamine:


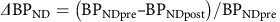


### Statistical Analysis

Demographic differences between groups, and injected mass/activity, were analyzed using independent-samples *t*-tests (2-tailed). An omnibus mixed-model ANOVA tested BP_ND_ as a function of Scan (pre-amphetamine *vs* post-amphetamine), ROI (10 levels) and Group (HV, PG). For analysis of simple main effects for our hypotheses concerning opioid release, we calculated percentage changes in [^11^C]carfentanil BP_ND_ (%ΔBP_ND_) from pre- to post-amphetamine scans. The subjective responses to the amphetamine challenge were analyzed using mixed-model ANOVAs based on the change from baseline values, with Group (HV, PG) as a between-subjects factor and Time (60, 120, 180, 270 min) as a repeated-measures factor. Given the ordinal relationship in the within-subjects factor (Time), we tested the linear and quadratic terms. For correlational analyses, we calculated a summary Δscore for subjective responses based on the SAIRS and SSAI, from the subjective peak minus the baseline. We tested for correlations between BP_ND_, subjective effects, and plasma amphetamine concentrations, using the subjective Δscores and baseline BP_ND_ and regional %ΔBP_ND_. Associations between MOR BP_ND_ and impulsivity measures were verified through Pearson's r correlation test. Percentile bootstrap (1000 replications) was used to estimate 95% confidence intervals (CI) for the correlation coefficient (Fethney, 2010). All data were normally distributed as determined by visual inspection as well as using the Kolmogorov–Smirnov and Shapiro–Wilk tests for normality. All statistical comparisons were assessed using SPSS version 20.0; *p*<0.05 was accepted as a nominal level of statistical significance.

## RESULTS

### Pharmacokinetic Amphetamine Plasma Samples

At 3 h post dosing, just before the post-amphetamine scan, the mean plasma amphetamine concentrations reached a peak of 86.8±18.2 ng/ml (mean±SD) in HV and 87.2±10.3 ng/ml in PG, with no significant group differences in amphetamine absorption (*t*_21_=0.06, *p*=0.951). There were no significant correlations between amphetamine plasma concentrations and baseline [^11^C]carfentanil BP_ND_ or %ΔBP_ND_.

### Injected Mass and Radioactivity

In HV, the mean injected [^11^C]carfentanil mass for scan 1 was 1.02±0.52 μg and scan 2 was 1.08±0.52 μg (*t*_14_=−2.51, *p*=0.025). For PG, the mean injected [^11^C]carfentanil mass was 1.53±0.27 μg for scan 1 and 1.45±0.20 μg for scan 2 (*t*_13_=2.39, *p*=0.032). There were also significant differences between groups in mass_pre_ (*p*=0.003) and mass_post_ HV/PG (*p*=0.015). There were no significant differences in injected activity between or within groups (HV: pre 225.1±65.0 MBq, post 208.9±67.1 MBq; PG: pre 207.9±67.7 MBq; post 214.0±49.2 MBq). To rule out any mass effect, we confirmed there were no significant correlations between mass_pre_ and baseline [^11^C]carfentanil BP_ND_ (*p*=0.851) or mass_post_ and post-amphetamine [^11^C]carfentanil BP_ND_ (*p*=0.918).

### Clinical Variables

There were no differences between PG and HV in age, IQ, smoking, and alcohol consumption; however, PG scored higher on measures of depression and anxiety though none reached a clinical threshold (see Table 1).

### [^11^C]carfentanil Binding

**Figure 1:**
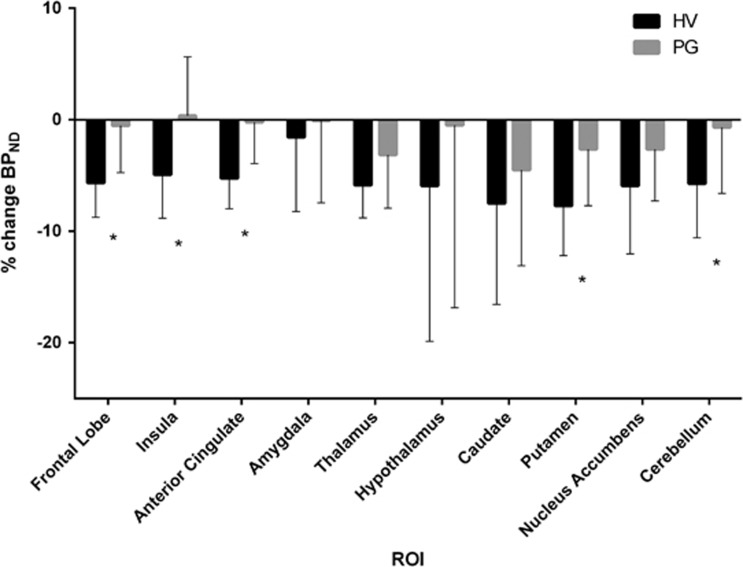
Percentage change (mean and SD) of [^11^C]carfentanil BP_ND_ from pre-amphetamine to post-amphetamine scan in HV and PG. There was a significant difference between groups in the frontal lobe, insula, anterior cingulate, putamen and cerebellum.

The omnibus ANOVA for BP_ND_ revealed a number of significant effects. There was a significant main effect of ROI (F(9,243)=248.5, *p*<0.001) indicating reliable differences in [^11^C]carfentanil binding across the 10 brain regions, with greatest binding in the nucleus accumbens and thalamus (see Table 2). There was a significant main effect of Scan (F(1,27)=26.4, *p*<0.001), and a Scan × ROI interaction (F(2.04,55.2)=3.07, *p*=0.053) consistent with amphetamine-induced opioid release that varied in extent across brain regions (see Figure 1). There was a significant Scan × Group interaction (F(1,27)=7.07, *p*=0.013), which is explored further below. The three-way Scan × Group × ROI interaction was not statistically significant (F(2.04,55.2)=0.37, *p*=0.696).

To directly test our hypotheses, first, we tested for group differences in baseline MOR levels using a Group × ROI model on the pre-amphetamine BP_ND_ levels. There was no significant main effect of Group (F(1,27)=0.01, *p*=0.923), or Group × ROI interaction (F(3.8, 103.8)=0.73, *p*=0.566). Thus, the hypothesis that PG would be associated with higher MOR levels was not supported.

Second, we confirmed that the amphetamine challenge led to significant reductions in [^11^C]carfentanil BP_ND_ in the HV group, using a Scan × ROI model. There was a significant main effect of Scan (F(1,14)=36.9, *p*<0.001), with no reliable Scan × ROI interaction (F(2.03,28.4)=2.18, *p*=0.132), driven by significant reductions in eight (caudate, putamen, thalamus, cerebellum, frontal lobe (includes dorsolateral, medial, and orbitofrontal cortices), nucleus accumbens, anterior cingulate, insular cortices of the 10 (amygdala, hypothalamus) regions of interest (see Table 2). Mean regional percentage reduction in BP_ND_ ranged between −4.9 and −7.7% (see Figure 1). There were no regions where increased BP_ND_ was observed. A *post hoc* analysis showed no impact of the interval between first and second scan on the results of the intervention (*r*=−0.11, *p*=0.563), and no difference between the HV BP_ND_ changes on same (*n*=10) and different (*n*=5) days (*t*=0.69, *p*=0.95). There were no differences between ‘past drug users' (>10 times in lifetime) and ‘non-past drug users' in either baseline [^11^C]carfentanil BP_ND_ (*t*=−0.97, *p*=0.34) or %ΔBP_ND_ (t=−0.24, *p*=0.98).

Third, to test whether PG was associated with blunted opioid release and decompose the Scan × Group interaction in the omnibus model, we ran a Group × ROI model on the %ΔBP_ND_ scores. There was a significant main effect of Group (F(1,27)=8.31, *p*=0.008) without a reliable Group × ROI interaction (F(1.9,50.7)=0.36, *p*=0.684). As such, the blunting of opioid release did not vary reliably across the 10 brain regions. The PG group showed significantly attenuated opioid release in the putamen (*t*_27_=2.85, *p*=0.008), cerebellum (*t*_27_=2.51, *p*=0.018), frontal lobe (*t*_27_=3.76, *p*=0.001), anterior cingulate (*t*_27_=4.17, *p*<0.001) and insula (*t*_27_=3.08, *p*=0.005).

### Effects of Amphetamine on Subjective Responses

**Figure 2:**
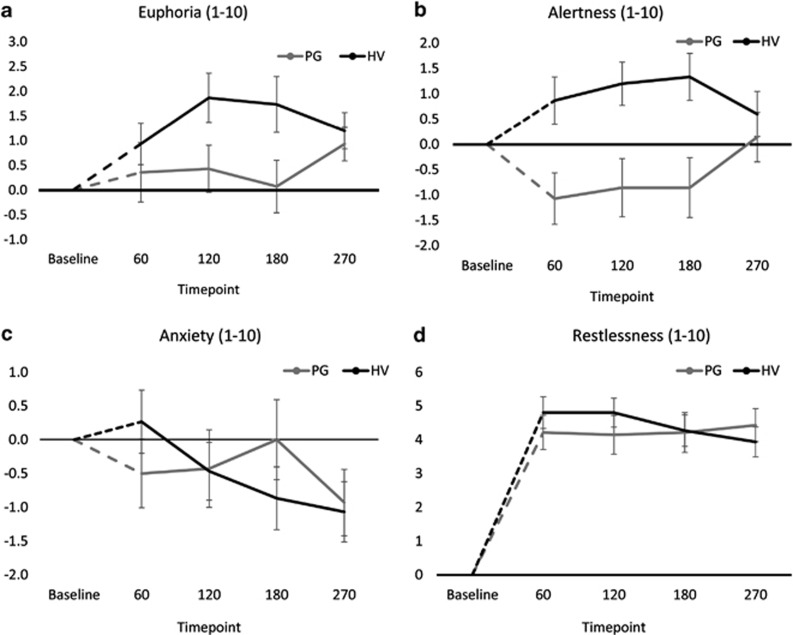
Changes in subjective amphetamine effects measured at four time points (minutes). (a) Amphetamine-induced euphoria was significantly diminished in the pathological gamblers group at 120 and 180 min. (b) For alertness scores, there were significantly diminished responses in the pathological gamblers group at 60, 120, and 180 min. (c, d) Groups did not differ significantly at any individual time point for anxiety or restlessness.

The subjective effects from the oral amphetamine were mild (see Figure 2). For euphoria ratings, an ANOVA of change from baseline values indicated no overall change in euphoria (main effect of Time: F(2.5,66.6)=1.09, *p*=0.353) but a significant quadratic term for the Group × Time interaction (F(1,27)=5.06, *p*=0.033), such that amphetamine-induced euphoria was diminished in the PG group at the 120 (*p*=0.047) and 180 (*p*=0.042) minute time points around the peak response (see Figure 2a). For alertness ratings, ANOVA yielded no overall change in alertness (main effect of Time: F(2.1,58.0)=0.86, *p*=0.435) but a significant Group × Time interaction (F(2.1,58.0)=3.54, *p*=0.032; see Figure 2b), with a diminished response in the PG group at 60 (*p*=0.009), 120 (*p*=0.007) and 180 (*p*=0.007) minutes. For anxiety ratings, there was an overall decrease in anxiety (F(3,81)=4.44, *p*=0.006) and a significant Group × Time interaction (F(3,81)=3.70, *p*=0.015) although groups did not differ significantly at any individual time point. There were no effects on restlessness (main effect of Time: F(1.8,49.5)=0.45, *p*=0.624; Group × Time F(1.8,49.5)=1.21, *p*=0.304).

### Relationship Between PET Measures and Clinical/ Impulsivity Scores

None of the clinical variables assessing severity of problem gambling (PGSI), craving to gamble (GACS), depression (BDI), anxiety (STAI, SSAI), alcohol use (AUDIT), or ‘days of abstinence' were significantly correlated with baseline [^11^C]carfentanil BP_ND_ or %ΔBP_ND_.

PG showed significantly higher scores in UPPS-P NU and PU subscales compared with HV (see Table 1). An exploratory analysis of HV and PG groups separately revealed a significant positive correlation between NU and baseline [^11^C]carfentanil BP_ND_ in the caudate in PG (*r*=0.638; *p*=0.014). There were no significant correlations with baseline BP_ND_ in HV.

## DISCUSSION

Using [^11^C]carfentanil PET, we demonstrate here evidence of blunted endogenous opioid release to an oral amphetamine challenge in PG compared with HV, whereas there was no difference in baseline MOR availability. Our hypotheses for this study were predicated on the view that as a behavioral addiction, PG would have similar neurobiological signature to that established for substance addictions. Our observation of blunted opioid release is consistent with the broader ‘reward deficiency hypothesis'. Our data are consistent with lower baseline endorphin levels reported in individuals with a positive family history of alcoholism compared with those with no family history, though increased endorphin release was seen after exposure to alcohol (Gianoulakis, 1996). Thus a dysregulated endorphin system appears to be present in behavioral and substance addictions. However, the hypothesis of higher baseline MOR availability in PG was not supported, in contrast to consistent findings in substance addiction. Similar to past work, we also found relationships between [^11^C]carfentanil BP_ND_ binding and trait impulsivity, but no relationships with other clinical variables.

Our key finding of a blunted release of endogenous opioid in PG following the oral amphetamine challenge strongly suggests a dysregulated opioid system in this disorder. Other studies measuring plasma *β*-endorphin during a gambling task are inconsistent with increase, no change, or blunted response reported (Blaszczynski *et al*, 1986; Meyer *et al*, 2004; Shinohara *et al*, 1999). The blunted opioid release in our study was accompanied by diminished subjective euphoria and alertness in PG in response to the amphetamine challenge, and was not explained by differences in plasma amphetamine levels between the groups. Another study has reported similarly increased euphoria in PG and controls following a similar oral amphetamine challenge to ours (0.4 *vs* 0.5 mg/kg) in combination with a dopaminergic PET tracer (Boileau *et al*, 2013). There are a number of pertinent differences that may underlie these contrasting effects. The Addiction Research Center Inventory used by Boileau assays a broader range of subjective responses than the SAIRS we used. In addition, our samples were all in treatment and had not gambled recently, whereas the participants in Boileau's study were non-treatment-seeking (Boileau *et al*, 2013). Another factor that may have moderated the response was that oral amphetamine was nonsalient as both PG and HV had limited or no experience of such stimulants. Thus our blunted response is consistent with other addiction studies, which have shown that responses to a nonsalient ‘reward' are blunted compared with salient ones (Lubman *et al*, 2008).

Concerning baseline MOR availability, our hypothesis that individuals with PG would show higher MOR availability was based on findings in individuals dependent on substances with differing pharmacologies, that is, cocaine (Zubieta *et al*, 1996), alcohol (Heinz *et al*, 2005; Williams *et al*, 2009), or heroin (Williams *et al*, 2007). Relationships have been described between higher MOR availability and greater craving in alcohol and cocaine addiction (Gorelick *et al*, 2005; Heinz *et al*, 2005; Williams *et al*, 2009; Zubieta *et al*, 1996). Such a relationship provides a potential mechanism for the clinical efficacy of opioid antagonists, naltrexone and nalmefene, in treating alcoholism (Lingford-Hughes *et al*, 2012). These medications have also shown efficacy in treating PG, which provides another rationale for predicting higher MOR availability (Ghitza *et al*, 2010). However, we did not observe any group difference in MOR availability or any relationship with craving in our PG sample. A key clinical variable that may impact on opioid receptor availability is abstinence. In cocaine addiction, higher levels of MOR availability are related to relapse and levels may reduce over time (Ghitza *et al*, 2010; Gorelick *et al*, 2008; Zubieta *et al*, 1996), though no changes in availability have been reported in alcoholism up to 3 months of sobriety (Heinz *et al*, 2005; Williams *et al*, 2009). Our PG were in treatment and abstinent at the time of scanning, so the influence of abstinence on MOR in behavioral addiction requires further investigation.

Consistent with previous research, we found significantly higher impulsivity scores using the UPPS scale in PG compared with HV (Clark *et al*, 2012; Forbush *et al*, 2008; Fuentes *et al*, 2006; Michalczuk *et al*, 2011). An exploratory analysis revealed that in PG, baseline [^11^C]carfentanil BP_ND_ in the caudate was positively correlated with the UPPS Negative Urgency subscale, which relates to the tendency towards impulsive behavior while experiencing negative affect (Whiteside and Lynam, 2001). This association was not observed in the HV group. These data also lend further support to a role of the endogenous opioid system in impulsive behaviors, particularly mood-related impulsivity, which is consistent with the proposed role for opioid system in emotion (Zubieta *et al*, 2003).

Although the efficacy of opiate antagonists in some people with PG was first reported over a decade ago, our study is the first to assess the integrity of the opioid system in PG, by imaging MOR availability and opioid release. Past work links the endogenous opioid system with pleasure, urges, and impulsivity (Love *et al*, 2009). For instance in HV, the opiate antagonist, naloxone, attenuated the fMRI response in the medial prefrontal cortex to monetary wins in a gambling task and increased responses to monetary losses in insula and anterior cingulate (Petrovic *et al*, 2008). The opiate antagonists, naltrexone and nalmefene were investigated as treatment for PG based on preclinical evidence of opioid involvement in urge and motivation and their efficacy in alcoholism (Grant *et al*, 2014; Potenza, 2008). A recent meta-analysis reported a small but significant effect of opiate antagonists though noted earlier studies were more likely to report efficacy (Bartley and Bloch, 2013). PG who respond to opioid antagonists report significant reduction in gambling urges, particularly in those with a family history of alcohol dependence (Grant *et al*, 2008; Potenza, 2008). In our sample, only one PG had a family history of alcoholism so we were unable to explore this further. The underlying mechanism for clinical efficacy of opioid antagonists in alcoholism is generally described as blocking the increased MOR and *β*-endorphin stimulation of MOR in mesolimbic dopaminergic pathway thus reducing activity and the rewarding effects of alcohol and craving (Johnson and North, 1992). Given our observation that MOR availability is unchanged in PG, the mechanism of action for opioid antagonists may therefore involve other opioid receptors such as kappa (Votinov *et al*, 2014) or processes other than those involved in pleasure and reward, such as impulsivity.

Dysregulation between opioid and dopamine transmission is likely to underlie our blunted opioid release in PG. Studies implicate a role for DRD2/3 in regulating endorphin release (Doron *et al*, 2006; Soderman and Unterwald, 2009). A role for the hypothalamus is probable since opioid projections originate from here to modulate dopaminergic neuronal activity in the ventral tegmental area (VTA) (Bourdy and Barrot, 2012). Concerning the dopaminergic system in PG, *increased* dopamine release in PG to a similar amphetamine challenge has been reported alongside no difference in dopamine receptor availability using [^11^C]PHNO PET (Boileau *et al*, 2013). In this study, [^11^C]PHNO binding in the hypothalamus was not assessed in PG and since we found increased [^11^C]PHNO binding in the hypothalamus in alcoholism with no differences elsewhere in the brain, it would be interesting to know if [^11^C]PHNO in the hypothalamus in PG was similarly increased (Erritzoe *et al*, 2014). Further investigation of dopamine–opioid interactions is warranted to characterize the sensitivity of dopaminergic system and whether there is reduced function of POMC-ergic hypothalamic neurons in PG.

In summary, we provide here the first evidence of a dysregulated opioid system in PG with blunted amphetamine-induced opioid release in the presence of normal MOR availability. The evidence from PET imaging of dopaminergic and opioid systems suggest that this behavioral addiction may differ from substance addiction with regard to receptor availability and release of endogenous neurotransmitters. Characterizing dopamine–opioid interactions will inform our understanding of substance and behavioral addictions as these neurotransmitter systems are critically involved. The reclassification of PG (and renaming to disordered gambling) in DSM-5 was based on evidence from epidemiological, clinical, and neurobiological data demonstrating similarities between PG and substance addiction (Clark and Limbrick-Oldfield, 2013). Therefore, further investigation of the neurobiology of PG with direct comparisons with other addictions is required to characterize their comparative neurobiology.

## Funding and Disclosure

This study was funded by the Medical Research Council- MRC G1002226. Anna Ramos has received financial support with a scholarship from CAPES (Process number: PDSE 99999.014476/2013-04). Dr Colasanti has been supported by a GSK/Wellcome Trust Fellowship in Translational Medicine and Therapeutics awarded through Imperial College London. The National Problem Gambling Clinic (Dr Bowden-Jones) receives some of its funding from the Responsible Gambling Trust. Dr Rabiner is a consultant for Lightlake Pharmaceutical, GSK, AbbVie, Teva, and a shareholder in GSK. Dr Waldman has received honoraria from Bayer, Novartis, and GSK, and has been a consultant for Bayer. Dr Stokes has received an honorarium from Indivior. Dr Clark has provided consultancy work for Cambridge Cognition Ltd, and the Centre for Gambling Research at UBC is funded by the Province of British Columbia and the British Columbia Lottery Corporation. Professor Gunn is a consultant for GSK, Abbvie, and UCB. Professor Lingford-Hughes has received research funding/support from Lundbeck, GSK, and honoraria for talks from Lundbeck. All the other authors report no conflict of interest.

## Figures and Tables

**Table 1:** Participants' Characteristics, Mean±SD

	**Healthy volunteers**	**Pathological gamblers**	**Significance (two-tailed)** ***p*-value**
Age	34.4±8.7	34.3±7.7	0.953
IQ	111.4±8.4	114.4±13.5	0.571
NART errors	19.2±7.2	17±10.8	0.595
PGSI	0.2±0.6	18±5.2	**0.001**
Alcohol units/week	6.8±8.5	11±7.7	0.254
Current smoking status	2 smokers	3 smokers	
Pack years (mean±SD)	14.25±5.7	14.5±5.3	0.963
FTND	2.5±3.5	6±1	0.386
Cigarettes/day	10±7.1	18±3.5	0.176
BDI on PET day	0.6±1.8	8.7±7.9	**0.002**
STAI	29.1±6.9	45.6±12.4	**0.001**
SSAI baseline	25.5±6.5	39.1±16.8	**0.012**
UPPS-NU	20.4±5.4	32.3±5.9	**0.001**
UPPS-PU	19.7±6.5	28.3±8.6	**0.018**
UPPS-LoP	20.3±5.7	23.9±5.5	0.155
UPPS-LoPe	18.4±4.8	20.2±4.6	0.387
UPPS-SS	33.3±9.7	35.1±7.0	0.622

Abbreviations: BDI, Beck Depression Inventory; FTND, Fagerstrom test for nicotine dependence; IQ, intelligence quotient; LoP, lack of premeditation; LoPe, lack of perseverance; NART, national adult reading test; NU, negative urgency; PGSI, Canadian problem gambling Inventory; PU, positive urgency; SS, sensation seeking; SSAI, Spielberger state anxiety inventory; STAI, Spielberger trait anxiety inventory; UPPS, impulsive behavior scale.

Questionnaire data were only available for nine HV participants, except for SSAI.Bold values indicate significant *p*-value.

**Table 2:** [^11^C]carfentanil BPND Pre- and Post-Amphetamine in the 10 Regions of Interest in the Healthy Volunteers (HV) and Pathological Gamblers (PG)

**HV: brain region**	**Mean pre-amph**	**Mean post-amph**	**Mean diff**	**SD mean diff**	**Mean % decrease**	**SD Mean % decrease**	**Sig (two-tailed)*p*-value**
Frontal lobe	1.13	1.06	0.07	0.04	−5.7	3.1	**0.001**
Insula	1.42	1.36	0.06	0.06	−4.9	3.9	**0.001**
Ant cingulate	1.49	1.41	0.08	0.04	−5.2	2.7	**0.001**
Amygdala	1.73	1.70	0.03	0.12	−1.5	6.7	0.335
Thalamus	2.03	1.91	0.12	0.06	−5.9	3.0	**0.001**
Hypothalamus	1.75	1.65	0.10	0.25	−5.9	14.0	0.121
Caudate	1.40	1.30	0.10	0.14	−7.5	9.1	**0.009**
Putamen	1.82	1.68	0.14	0.08	−7.7	4.5	**0.001**
Accumbens	2.76	2.60	0.16	0.18	−5.9	6.1	**0.004**
Cerebellum	0.73	0.69	0.04	0.03	−5.7	4.9	**0.001**

**PG: brain region**	**Mean pre-amph**	**Mean post-amph**	**Mean diff**	**SD Mean diff**	**Mean % decrease**	**SD Mean % decrease**	**Sig (two-tailed)*p*-value**
Frontal lobe	1.03	1.02	0.01	0.04	−0.5	4.2	0.585
Insula	1.39	1.39	0.00	0.07	0.3	5.3	0.858
Ant cingulate	1.43	1.43	0.00	0.05	−0.2	3.7	0.795
Amygdala	1.82	1.82	0.00	0.14	−0.1	7.4	0.817
Thalamus	2.07	2.01	0.06	0.10	−3.1	4.8	**0.029**
Hypothalamus	1.78	1.76	0.02	0.24	−0.5	16.4	0.769
Caudate	1.39	1.33	0.06	0.11	−4.5	8.6	0.058
Putamen	1.78	1.70	0.08	0.09	−2.7	5.1	0.071
Accumbens	2.78	2.71	0.07	0.13	−2.6	4.7	0.056
Cerebellum	0.77	0.77	0.00	0.05	−0.7	5.9	0.675

Bold values indicate significant *p*-value.
